# *Pulsatilla chinensis* (Bge.) Regel: A Systematic Review on Anticancer of Its Pharmacological Properties, Clinical Researches and Pharmacokinetic Studies

**DOI:** 10.3389/fonc.2022.888075

**Published:** 2022-06-23

**Authors:** Hang Li, Lilan Wang, Xiaojing Zhang, Wenxin Xia, Xirong Zhou, Hong Sui, Xueyan Fu

**Affiliations:** ^1^ School of Pharmacy, Ningxia Medical University, Yinchuan, China; ^2^ Ningxia Minority Medicine Modernization Key Laboratory of Ministry of Education, Yinchuan, China

**Keywords:** Pulsatilla chinensis (Bge.) Regel, anti-tumor, molecular mechanism, clinical researches, pharmacokinetic studies, bioinformatics research

## Abstract

Pulsatilla chinensis (Bge.) Regel (PC) is one of the most commonly used Chinese medicines and has a history of thousands of years. This article reviews the research results of anti-cancer activity and its mechanism of action obtained from experimental, clinical, pharmacokinetic and bioinformatic studies in recent years. A large number of studies have shown that PC exerts had anti-cancer effects on different types of tumor cells by inhibiting cell proliferation, inducing apoptosis, inhibiting cell cycle and energy metabolism, inducing autophagy, and inhibiting angiogenesis. The literature has shown that PC can trigger the expression of autophagy-related molecules, activate the mitochondrial apoptotic pathway, inhibit the phosphorylation of PI3K downstream factors, down-regulate the expression of glycolysis-related proteins, and regulate a series of cancer-related signal pathways and proteins. The molecular mechanisms involved in PC include signal pathways such as Notch, PI3K/AKT/m TOR, AKT/mTOR, and MEK/ERK. The article also discusses the derivatives of the active ingredients in PC, which greatly improved the anti-cancer effect. In conclusion, this review provides a comprehensive overview of the biological effects and mechanisms of PC against cancer. The analysis of the literature shows that PC can be used as a potential drug candidate for the treatment of cancer.

**Graphical Abstract:**
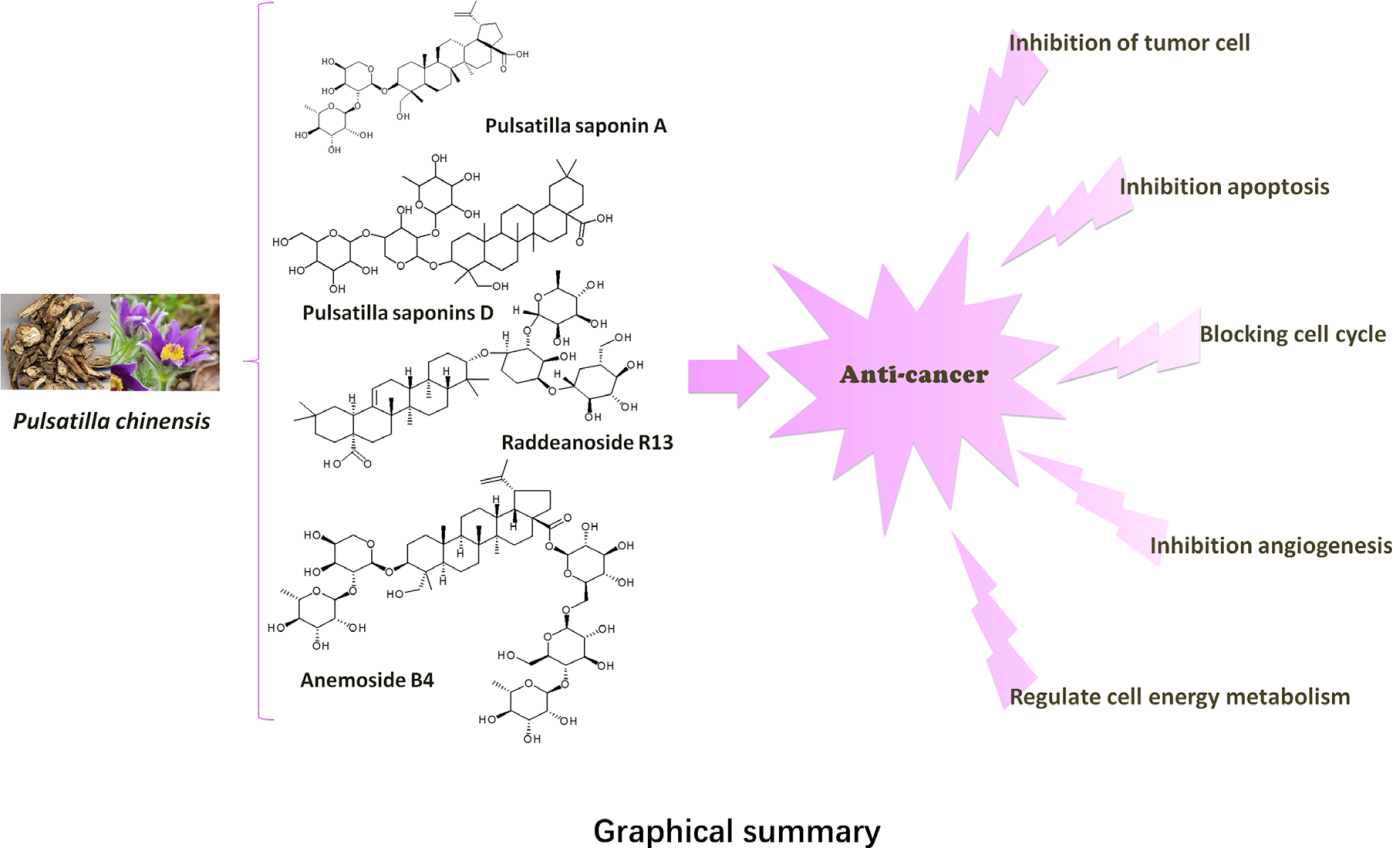


## Retrieval Method

Information on the studies of *Pulsatilla chinensis* (Bge.) Regel (PC) is collected from scientific journals and reports *via* library and electronic data search (PubMed, Baidu Academic, Google Scholar, Science Direct, ACS, Web of Science, and CNKI). Meanwhile, it is also obtained from published works of folk records, ethnopharmacological literature, Ph.D. and Master’s Dissertation. The specific search results are as follows.

The keywords of the second part are “chemical composition of PC”. A total of 32 pieces of literature were retrieved. The keywords of the third part are “PC anti-tumor”, “PSA anti-tumor”, “AB4 anti-tumor”, and “PSA anti-tumor”. A total of 79 pieces of literature were retrieved. After the screening, 62 kinds of literature met the requirements. The keywords of the fourth part are “anti-tumor of PC” and “clinical application of PC”. A total of 7 literature were retrieved. 3 literature were screened to meet the requirements. The keywords of the fifth part are “PC pharmacokinetics”. A total of 11 pieces of literature were retrieved. The keywords of the sixth part are “network pharmacology of PC”, “modern technology of PC”, and “molecular docking technology of PC”. A total of 3 literature were retrieved.

## Introduction

Traditional Chinese medicine (TCM) has been an integral part of healthcare in China for thousands of years. In modern medicine, Chinese herbs also have been used to treat a variety of diseases. For example, the anti-cancer effect of Pulsatilla chinensis (Bge.) Regel (PC) had been found in previous research studies. PC is a common TCM, which belongs to the plant genus Pulsatilla of the Ranunculaceae family. Because PC likes to be born in fields, wetlands, riverbanks, and damp grasses, it is widely distributed in China, such as Sichuan, Hubei, Jiangsu, Jilin, Heilongjiang, etc. In addition, PC has also been found in North Korea and the Russian Far East.

The first description of PC was recorded in the Shijing as early as the Western Zhou Dynasty. In addition, PC was first documented as a medicinal material in Shen Nong’s Herbal Classic, recording that it tasted bitter and cold. It is mainly used for treating malaria fever, dispelling dampness and heat, cooling blood, relieving pain, and treating malignant sores. Among them, concretions and gatherings were referred to as a type of tumor in modern medicine.

In recent years, a large number of literatures have reported that the pentacyclic triterpenoid saponins in PC (PC saponins) have significant anti-tumor activities *in vitro* and *in vivo*, such as inhibition of cell proliferation, effects of signal transduction, apoptosis and tumor invasion, etc (1). Modern pharmacological studies have shown that a large number of researchers have conducted extensive research on PC saponins, for their cancer chemopreventive potential against various cancers, for instance, gastric cancer, lung cancer, breast cancer, cervical cancer, hepatocellular carcinoma, pancreatic cancer (2), leukemia, colon cancer, multiple myeloma, etc (3). In particular, pulsatilla saponin A (PSA) (4), Anemoside B4 (AB4) (5), raddeanoside R13 (R13) (6), and Pulsatilla saponins D (PSD) (7) proved to have strong anti-tumor effects too. At the same time, it has also been widely studied.

As people pay more and more attention to the anti-cancer effects of PC, a lot of researchers had been conducted on its pharmacological effects and clinical applications. However, the literature lacked a systematic and comprehensive review of the anti-tumor effects of PC. Considering the complexity of TCM, more scientific research was needed to determine its chemical composition, biological activity, and potential molecular mechanism. In this review article, the mechanisms and biological effects of PC were reviewed from seven aspects: cell proliferation, apoptosis, cell cycle, energy metabolism, drug resistance, autophagy, and angiogenesis. And through the evaluation of its curative effect and therapeutic use, its clinical application value was discussed. This article also discussed the pharmacokinetic and bioinformatic studies of PC and proposes future research directions, to make this review article a useful reference resource for researchers.

## Chemical Ingredients

**Figure 1:**
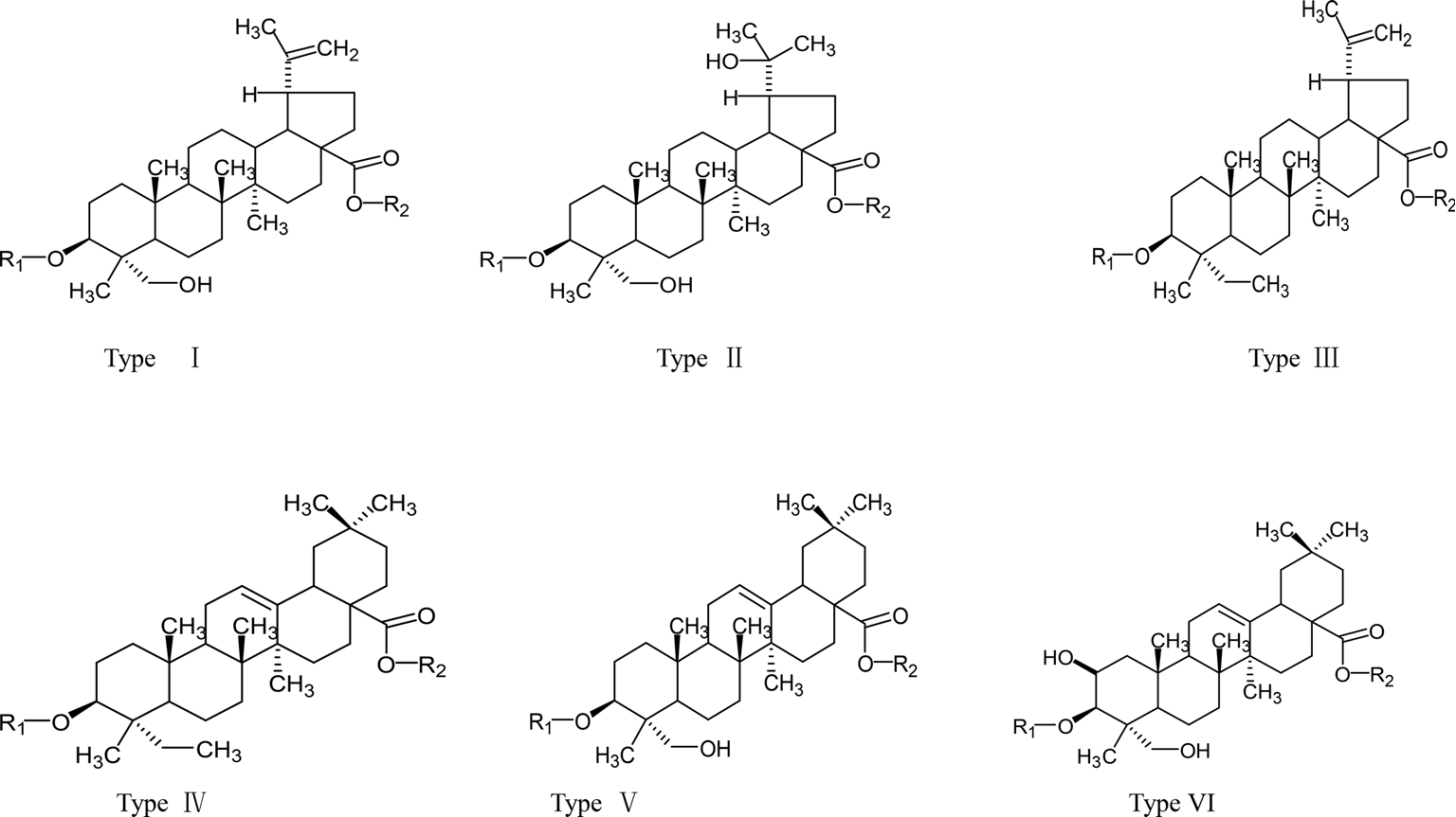
The types of *Pulsatilla chinensis* (Bge.) Regel aglycones.

In recent years, more and more scientists have paid attention to the bioactive molecules and anti-tumor activity of PC. Early literature reported that PC contained Anemonin, Okinalin, and Okinalein, etc (8). Subsequently, triterpene acids, lignans, carotinosides (9), non-O-linked glycoprotein components PCG-A (10), and a toxic protein AME (11) were gradually isolated from PC. According to the literature, the anti-tumor components of PC were mainly pentacyclic triterpenoid saponins. Chen Wenkan (12, 13), Yoshihirol (14, 15), etc. respectively extracted and separated various lupinane triterpenoid saponins and oleanane triterpenoid saponins from PC. And their aglycones were mainly divided into three types: oleanolic acid sapogenin, ivy sapogenin, and 2,3-hydroxy betulinic acid sapogenin (16). The names of these constituents were listed in **Table 1**, and their chemical structures were shown in **Figure 1**. In addition, the structure-activity relationship indicated that oleanane-type saponins had better cytotoxic activity than lupinane-type saponins. This was inseparable from the free carboxyl group on the aglycon C-28. Similarly, the length and bonding degree of the ethanol chain on the aglycon C-3 also had an important influence on the cytotoxic activity (30).

**Table 1:** Types of saponins in Pulsatilla chinensis (Bge.) Regel.

Aglycon structure	R1	R2	Compound	References
I	ara	H	3 - O - alpha - L - pyran Arabian sugar - 3 beta, 23 - dihydroxy lupine alkanes - Δ ^20 (29)^ ene - 28 - acid	(14)
IV	ara	H	Oleanolic acid -3-O- -L- pyranoid arabinoglycoside	(17)
IV	ara	H	Ivy-saponin - 3- O -L- pyranarabinoglycoside	(15)
V	ara(Connected to C23)	H	Ivy-saponin -23- O -L-pyranoid arabinoglycoside	(7)
I	rha(1→2)ara	H	3 - O - alpha - L - pyran rat sugar-based li - (1, 2) - alpha L - pyran Arab sugar-based - 3 beta, 23 - dihydroxy lupine alkanes - Δ ^20 (29)^ ene - 28 - acid	(17)
I	H	glc(1→6)glc	3 beta, 23 - dihydroxy lupine alkanes - Δ ^20 (29)^ ene - 28 - O - beta - D - pyran glucose - 6 (1) - beta - D - pyran Portugal also glycoside	(18)
I	glc(1→4)ara	H	3 - O - beta - D - glucose base - (1, 4) - pyran alpha L - pyran Arab sugar-based - 3 beta, 23 - dihydroxy lupine alkanes - Δ ^20 (29)^ - ene - 28 - acid	(19)
III	rha(1→2)ara	H	3 - O - alpha - L - pyran rat lee sugar - (1, 2) - alpha - L - pyran Arab sugar-based - lupine alkanes - Δ ^20 (29)^ ene - 28 - acid	(14)
IV	rha(1→2)ara	H	Oleanolic acid, 3-O -L-rhamnoose - (1, 2)- -L-pyran arabinoglycoside	(14)
IV	H	glc(1→6)ara	Oleanolic acid, 3-O -L-rhamnoose - (1, 2)- -L-pyran arabinoglycoside	(17)
IV	rha(1→2)ara	H	Ivy-saponin 3-O - -L-rhamnoose - (1, 2)- -L-arabinoglycoside	(20)
IV	glc(1→4)ara	H	Saponin 3-O- D- pyranoid glucosaccharide - (1, 4) -L- pyranoid arabinoglycoside	(17)
V	glc(1→2)ara	H	Ivy-saponin 3-O - D- pyranosinose - (1, 2)- -L-arabinoglycoside	(21)
I	H	rha(1→4)glc(1→6)glc	pulsatilla saponin C	(22)
I	rha(1→2)[glc(1→4)]ara	H	3 - O - alpha - L - pyran rat lee sugar - (1, 2) - [beta - D - pyran glucose - (1, 4)] - alpha - L - pyran Arabia candy - alkanes feather fan beans - Δ ^20 (29)^ - ene - 28 - acid	(19)
III	glc(1→3)rha(1→2)ara	H	3 - O - beta - D - glucose base - (1, 3) - pyran alpha L - pyran rat sugar-based li - (1, 2) - alpha - L - pyran Arab sugar-based - lupine alkanes - Δ ^20 (29)^ - ene - 28 - acid	(17)
III	glc(1→2)rha(1→2)ara	H	Oleanolic acid 3-O - -D- pyran glucose - (1 3)- -L-pyran rhamnoose - (1 2)- -L-pyran arabinoglycoside	(19)
III	rha(1→2)glc(1→4)ara	H	Oleanolic acid 3-O -L-rhamnoose - (1 2)- -D-glucose-pyranoid - (1 4)- -L-arabinoglycoside	(23)
IV	ara(1→3)[rha(1→2)]ara	H	Oleanolic acid 3 - O - alpha - L - pyran Arabia candy - (1, 3) - [alpha - L - pyran rat lee sugar - (1, 2)] - beta - D - pyran arabinoside	(24)
IV	glc(1→3)rha(1→2)ara	H	Ivy-saponin 3-O - D- pyranosaccharide - (1 3)- -L-pyranosaccharide - (1 2)- -L-pyranosaccharide	(24)
IV	rha(1→2)glc(1→4)ara	H	Ivy-saponin 3-O – L-rhamnoose - (1 2)- -D-glucose-pyranoid - (1 4)- -L-arabinoglycoside	(23)
V	ara(1→3)[rha(1→2)]ara	H	Saponin 3-O - L-pyran arabinoglycoside - (1)-[-L-pyran rhamnoglycosyl - (1 2)]- -D-arabinoglycoside	(20)
V	H	rha(1→4)glc(1→6)glc	Ivy-saponin 28-O -L-Rhamnoose - (1 4)- -D-glucose-pyranoid - (1 6)- -D-glucosinopyranoid	(24)
V	rha(1→2)[glc(1→4)]ara	H	pulsatilla saponin D	(20)
IV	H	rha(1→4)glc(1→6)glc	2 -hydroxyglucoside 28-O- α-L- Rhamnoose pyranoid - (1 4)- β-D-glucose-pyranoid - (1 6)- β-D-glucose-pyranoid	(14)
I	ara	rha(1→4)glc(1→6)glc	Pulsatilla saponin B	(15)
III	glc(1→4)glc(1→3)rha(1→2)ara	H	Oleanolic acid 3-o - β-D-glucose-pyranoid - (1 4)-β -D-glucose-pyranoid - (1 3)-α -L-rhamnoose - (1 2)- α-L-arabinoglycoside	(25)
IV	rha(1→2)ara	glc(1→6)glc	3-O-α -L-pyranosyl - (1 2)-α -L-pyranosyl arabinoglycolic acid 28-O-β -D-glucoglucan - (1 6)-β -D-glucoglucan ester glycoside	(20)
IV	ara	rha(1→4)glc(1→6)glc	3- O-α -L- Pyranoid Glucoside 28-O- α-L- Pyranoid rhamnoose - (1 4)- β-D-glucoglucoside - (1 6)- β-D-glucoside	(26)
I	rha(1→2)ara	rha(1→4)glc(1→6)gl	Anemoside B4	(26)
I	glc(1→3)ara	rha(1→4)glc(1→6)gl	3 - O - alpha - L - pyran rat lee sugar - (1, 2) - alpha L - pyran Arabia candy - 3 beta hydroxy - Δ ^20 (29)^ ene - lupine alkanes - 28 - O - alpha - L - pyran rat lee sugar - (1, 4) - beta - D - pyran glucose - 6 (1) - beta - D - pyran glucose ester glycosides	(27)
III	rha(1→2)ara	rha(1→4)glc(1→6)gl	3-o - α-L-pyranosaccharide - (1 2)- α-L-pyranosaccharide -3β,20, 23-trihydroxy-lupane -28-O-α -L-l-pyranosaccharide - (1 4)-β -D-glucopyranosaccharide - (1 6)- β-D-glucopyranosaccharide	(14)
II	rha(1→2)ara	rha(1→4)glc(1→6)gl	Oleanolic acid 3-O-β-D-glucopyranose-(1→4)-β-D-glucopyranose-(1→3)-α-L-rhamnose-(1→2))-β-D-glucopyranose-(1→4)-α-L-arabinopyranoside	(14)
II	glc(1→4)glc(1→3)rha(1→2)glc(1→4)ara	H	3-O-α-L-rhamnose-(1→2)-α-L-arabinose oleanolic acid 28-O-α-L-rhamnose-(1→4)-β-D-glucopyranoside-(1→6)-β-D-glucopyranoside	(27)
II	rha(1→2)ara	rha(1→4)glc(1→6)glc	Oleanolic acid 3-O-β-D-glucopyranose-(1→4)-β-D-glucopyranose-(1→3)-α-L-rhamnose-(1→2))-[β-D-glucopyranose-(1→4)]-α-L-arabinose	(15)
IV	glc(1→4)glc(1→3)rha(1→2)[glc(1→4)]ara	H	3-O-α-L-rhamnanopyranosyl-(1→2)-α-L-arabinose and ivy sapogenin 28-O-α-L-rhamnose-(1→4)-β-D-glucopyranoside-(1→6)-β-D-glucopyranoside	(27)
IV	rha(1→2)ara	rha(1→4)glc(1→6)glc	3-O-β-D-glucopyranose-(1→2)-β-D-glucopyranose ivy sapogenin 28-O-α-L-rhamnose-(1→4)-β -D-glucopyranose-(1→6)-β-D-glucopyranose glycoside	(28)
V	rha(1→4)glc(1→6)glc	rha(1→4)glc(1→6)glc	2-O-β-D-glucopyranose-(1→2)-β-D-glucopyranose ivy sapogenin 28-O-α-L-rhamnose-(1→2)-β -D-glucopyranose-(1→6)-β-D-glucopyranose glycoside	(28)
I	rha(1→2)[glc(1→4)]ara	rha(1→4)glc(1→6)glc	Pulsatilla saponin E	(26)
IV	rha(1→2)[glc(1→4)]ara	rha(1→4)glc(1→6)glc	3-O-α-L-rhamnose-(1→2)-[β-D-glucopyranose-(1→4)]-α-L-arabinose ivy sapogenin 28- O-α-L-rhamnose-(1→4)-β-D-glucopyranose-(1→6)-β-D-glucopyranoside	(29)
IV	glc(1→3)rha(1→2)ara	rha(1→4)glc(1→6)glc	3-O-β-D-glucopyranose-(1→3)-α-L-rhamnose pyranos-(1→2)-α-L-arabinose ivy sapogenin 28-O- α-L-rhamnose-(1→4)-β-D-glucopyranose-(1→6)-β-D-glucopyranose glycoside	(29)
I	glc(1→4)glc(1→4)[rha(1→2)]ara	rha(1→4)glc(1→6)glc	3-O-β-D- Glucopyranose-(1→4)-β-D- Glucopyranose-(1→4)-[α-L- Rhamnose-(1→2)]- α-L-arabinose oleanolic acid 28-O-α-L-rhamnose-(1→4)-β-D-glucopyranose-(1→6)-β-D- Glucopyranose	(14)

Some studies showed that Pulsatilla saponin D (PSD) and Pulsatilla saponin A (PSA) had the strongest anti-tumor activity among PC saponins, and their IC_50_ for NCI-H460 cells were 5.2, and 7.9 μg/mL, respectively (31). Among these biologically active molecules, the release of the carboxyl group at position C-28 of PSD was essential for the anti-tumor activity of saponins. At the same time, the type of aglycon, the number of sugar groups, and the sugar chain sequence were rha(1→2)[glc(1→4)ara saponin, which had strong anti-tumor activity (32). Notably, to better apply the active compounds of PC saponins in clinics, PSA and PSD derivatives with C ring or C-28 or C-3 modifications were synthesized (32). In addition to PSD and PSA, raddeanoside R13 (R13) had clear anti-tumor activity and their IC_50_ for NCI-H460 cells was 4.6 μg/mL.

Among the PC, anemoside B4 (AB4) had the highest content in the herb, and it was used as a quality control marker for the PC, quantified as over 4.6% (33). At present, AB4 is the most studied active compound in PC. And some researchers reviewed the pharmacological effects of AB4 (34). However, the anti-tumor effect of AB4 had not been elucidated in detail. Particularly, studies have confirmed that AB4 can reduce the toxicity of cisplatin. After 10 days of intraperitoneal injection at a dose of 3 mg/kg, the inhibitory rate of AB4 (98.4%) on tumor cells was greater than that of cisplatin (95.1%) (35). This will provide a direction for the future anti-tumor research of AB4.

## Anti−Tumor Effects

**Figure 2:**
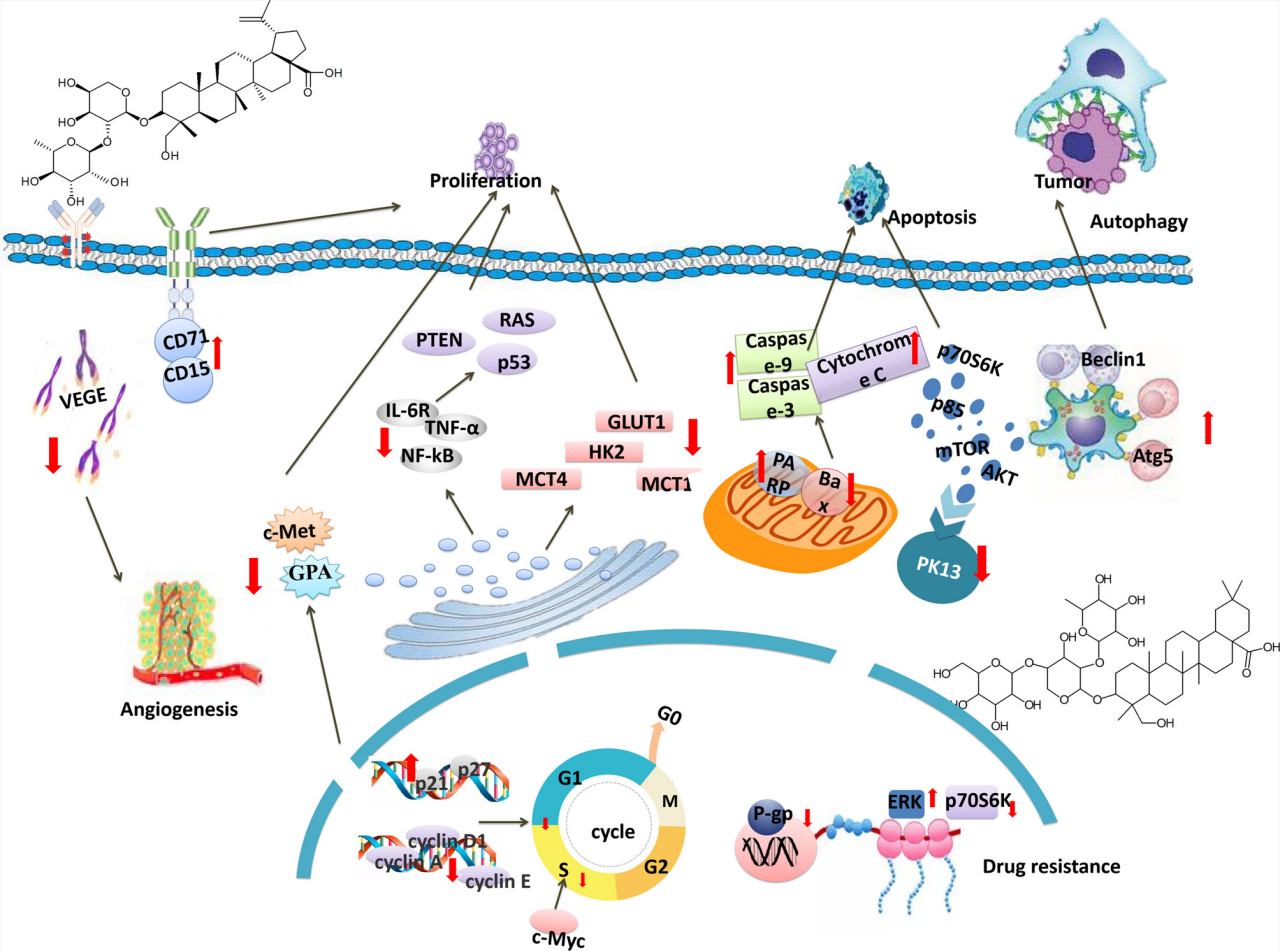
Molecular mechanism of Plusatilla chinensis (Bge.) Regel.

This article provides a comprehensive review of the literature on the anticancer activity of PC. The anti-tumor mechanism of PC is mainly to inhibit cell proliferation, induce apoptosis, inhibit cell cycle, regulate cell energy metabolism, reverse drug resistance, induce autophagy and inhibit angiogenesis. The specific molecular pathways are shown in **Table 2** and **Figure 2**.

**Table 2:** The studies of Pulsatilla chinensis (Bge.) Regel. on different cancer cell lines and normal cell lines.

Serial number	References	Compound	Inhibitory concentration	Cell Line	Improved characteristics
Inhibition of tumor cell					
1	(36)	PSD	6.4 mg/kg	LLC cells	inhibition rate 82%
			9 μg/m L	SW480	Down-regulating proteins GLUT1, HK2, MCT4, MCT1, c-Myc, HIF-1a mRNA↓
2	(37)	PSD	5.2μg/mL(IC_50_)	NCI-H460	Reduced expression of ERK1/2, Ras, GLUT1, MCT4 protein↓
		R13	4.6μg/mL(IC_50_)		
3	(38)	PSA	7.9μg/mL(IC_50_)		
4	(39)	PSD	3.76 μg/mL(IC_50_)	HT-29	
5	(40)	PSD	5 and 10 µM	MIAPaCa-2, BXPC-3, PANC-1, AsPC-1 and HPAC	increasing levels of cleaved caspase-3 and decreased Bcl-2 expression *via* mitochondrial membrane potential.↑
6	(38)	PSA	10.56 ± 0.68μg/ml(IC_50_)	H1975	
7	(41)	PC saponins	100 mg/kg	LLC	down-regulating the expression level of IL-6R, TNF - α, NF-kB and regulate the expression of RAS, c-myc, p53, PTEN↓
8	(42)	PC saponins	1.440μg/ml(IC_50_)	HT29	*the expression levels of calpain1 and N-cadherin decreased*↓*, the expression levels of E-cadherin increased*↑
9	(43)	PC saponins	1,2,4,8,12 mg/L	CAL27	Decreasing calpain1, N-cadherin↓ increasing E-cadherin↑
10	(44)	PC saponins	3.44-9.91μg/ml(IC_50_)	BGC-823、SGC-7901、NCI-H460、A549、SMMC-7721、Bel-7402、	LC3-I is decomposed into autophagosome membrane type LC3-IIand reduces the content of HK II, PFK
			none	HT-29、HCT116、U251、SK-0V-3、PC-3and K562	and PK↓
11	(40)	PSD	4.5, 6.0 mg/kg	LLC	tumor inhibition rates are 38.67% and 41.59%
12	(45)	PSD	50 nmol/ml	MCF-7	Inhibition the activation of Wnt/β-chain protein signaling pathway and inhibition the expression of downstream proteins Cyclin D1 and c-Myc↓
13	(46)	PSD	20,50,100 nmol/m L	HeLa	
14	(47)	AB4	50 μmol/L	Huh-7	Decreased expression of Bcl-2 protein↓ and increased expression of Bax, Caspase-3, Cleaved Caspase-3, Cleaved PARP protein↑
15	(48) (49)	PSA	none	K562	up-regulating the expression of CD71 ↑
					and GPA differentiation antigens on the surface ↑
16	(50)	PSA	5, 4, 3μg/ml	K562, U937 and HL-60	Up-regulating the phosphorylation level of ERK and the expression of CD15 differentiation antigen↑.
17	(51)	AB4	8, 16, 32 μmol/L	Burkitt lymphoma cells	Bcl-2 protein expression decreased↓, Bax Cleaved PARP Cleaved Caspase-3 protein expression increased ↑. The protein expression of P-JAK2 and P-STAT3 decreased↓.
18	(52)	AB4	45.05 ± 1.77 μmol/L(IC_50_)	Hep G2	Increasing the expression of apoptotic proteins Cleaved Caspase-3, Cleaved PARP, Cytochrome C↑
19	(36)	AB4	20mg/mL	HeLa	Inhibition Notch signaling pathway↓ and promoting the expression of autophagy-related molecules Beclin1 and Atg5↑
Inhibition apoptosis					
20	(53)	PC saponin	5.6µM(IC_50_)	Hep-7402	LDHA, PI3K, NOL3, and cleaved-caspase-3 may play a major regulatory role
21	(54)	PSD	5.9µM(IC_50_)	NCI-H460	Down-regulate BCL-2 and caspase-3 signal transduction↓
		R13	5.1µM(IC_50_)		Down-regulate the expression of proteins PI3K, p-Akt, p-m TOR, and p-p70S6K↓
		PSA	10.5µM(IC_50_)		
22	(55)	PSD	20 40 μmol/L	A549	Induction of expression of Bax and cleaved caspase-3
23	(56)	PSD	25.00μg/m L	BEL-7402	Up-regulating the expression of Caspase-3↑ and down-regulating the expression of Bcl-2↓
24	(57)	PC saponins		SCC-25	
25	(58, 59)	PSD	15.0, 20.0, 25.0μmol/L	McF-7	Down-regulating the expression of PI3K/AKT/mTOR signaling pathway related proteins PI3K-p85, p-AKT, p-mTOR, p-p70S6K protein↓
26	(60)	PC saponin	8ng/ul(IC_50_)	U266	Up-regulating the expression of CD49e↑
Blocking cell cycle					
27	(61)	AB4	40 mg/mL	HepG2	Blocking G2/M phase replacement
28	(62)	PC saponins	6.25, 12.50, 25.00μg/ml	SMMC-7721	Blocking S phase
				A549	Blocking G0/G1
Inhibition of tumor angiogenesis					
29	(63)	R13	0.54-0.86 ×10^-5^mol/L(IC_50_)	ZR75-1and MCF-7	Inhibition G1/S phase
30	(64)	PSD		HCC	decreasing the expression of HIF-1α and its target gene VEGF↓
31	(65)	PSD	4μg/ml	gastric cells	inhibiting expression of activated mesenchymal-epithelial transition factor (c-Met)↓
Regulate cell energy metabolism					
32	(66)	PC saponins	1-9 μg/m L	SW480	Inhibition HIF-1α protein expression and down-regulating HK-II, PKM2 and GLUT1 mRNA↓
33	(67)	PC saponins	11.69μg/mL(IC_50_)	SW480	Decreasing c-Myc, HIF-1a protein and lactate transporter MCT4↓
Reversal of drug resistance					
34	(68)	AB4	0.45μg/m L	L-OHP	P-gp gene expression decreased in drug-resistant cells↓
35	(69)	PSD	none	HeLa	increasing the phosphorylation of ERK↑ and inhibiting the phosphorylation of mTOR and p70S6K↓
Inducing autophagy in tumor cells					
36	(70)	PSD	25μg/m L	MCF-7 and MDA-MB-231	the autophagosome-lysosome fusion was blocked
37	(71)	AB4		SMMC7721	Acting on the Bcl-2-caspase-3 pathway and Beclin-1-LC3-p62 pathway
38	(72)	AB4	20、40、80μM	SMMC7721	Up-regulating the expression of LC3-II/LC3-I, Beclin-1 protein↑.Down-regulating p62 protein and PI3K/Akt/mTOR signaling pathway p-Akt/Akt, p-mTOR/mTOR↓

↑, Up-regulating; ↓, Down-regulating; AB4, Anemoside B4;PSD, Pulsatilla saponin D;PSA, Pulsatilla saponin A; PC saponins, Pulsatilla chinensis (Bge.) Regel. saponins.

### Inhibition of Tumor Cell Growth and Proliferation

More and more TCM plays a role in different stages of tumorigenesis (73). Mechanistically, these treatments inhibit the synthesis of DNA, RNA, and proteins and thus inhibit the proliferation of tumor cells (74). Next, we will discuss the important mechanism of PC extract anti-tumor and the tumor inhibition rate at different doses.

#### Pulsatilla saponin D

A previous study had shown PSD had a potent inhibition rate of tumor growth (IR, 82%) at the dose of 6.4 mg/kg on the BDF1 mice bearing LLC cells (36). Similarly, PSD had the strongest effect on human colorectal adenocarcinoma cell HT-29 (IC_50_ = 3.76 μg/mL). It is reported that PSD can achieve an anti-tumor effect by enhancing the expression of Caspase3 protein and LC3-II protein (37). Moreover, the investigation found that the anti-proliferative effect of PSD was demonstrated by increasing levels of cleaved caspase-3 and decreasing Bcl-2 expression *via* mitochondrial membrane potential, as well as elevating numbers of terminal deoxynucleotidyl-transferase-mediated dUTP nick end labeling (TUNEL) (39). Admittedly, PSD, at doses of 5 and 10 µM, strongly inhibited up to 80% of cell growth in a dose-dependent manner. Further exploration showed PSD (4.5, 6.0 mg/kg) had a significant inhibitory effect on the H22 hepatoma solid tumor in mice, and the tumor inhibition rates were 38.67% and 41.59%, respectively. It was found that PSD could increase the expression of Wnt protein in tumor tissues, and decrease the relative expression of C-Myc and β-catenin proteins (40). In addition, it had been suggested that PSD inhibited the abnormal activation of the Wnt, which led to the timely degradation of β-catenin, thereby inhibiting the downstream gene cyclin D1and cMyc (46). Therefore, the results of this experiment suggest that PSD had a significant inhibitory effect on cell proliferation. The mechanism may be related to inhibiting the activation of the Wnt/β-chain protein signaling pathway (45).

#### Pulsatilla Saponin A

PSA can be combined with 5-fluorouracil or used alone. The effect showed that protein 53 and cleaved caspase 9 were increased. At the same time, B-cell lymphoma 2 protein expressions were decreased (38). PSA can promote the overexpression of miR-24-3p, and then target the down-regulation of RNF2 expression to inhibit tumor cells. Therefore, PSA may influence the proliferation of cancer cells through the Mir-24-3p/RNF2 pathway (75). In addition, recent studies have shown that PSA also regulated JAK2/STAT3 signaling pathway to inhibit Burkitt lymphoma cell proliferation (76).

In addition, PSA can also inhibit the proliferation of tumor cells in combination with chemotherapy. PSA significantly increased the killing and growth inhibitory effects on non-small cell lung cancer H1975 cells(IC_50 =_ 10.56 ± 0.68μg/ml). A study showed that PSA combined with ionizing radiation can reduce the expression of DNA damage repair-related proteins mre11, DNA-pkcs, ku80, cycle-related proteins cycline, cyclinb1, and anti-apoptosis related protein BCL-2 (77). Simultaneously, PSA can also down-regulate SIRT1 protein to increase chemotherapy sensitivity (78).

Currently, PSA has been reported to induce tumor cell differentiation and inhibit its proliferation. A study showed that PSA can increase the hemoglobin content in K562 cells. This mechanism was related to the up-regulation of the expression of CD71 and GPA differentiation antigens on the cell membrane surface (48). In addition, acute myeloid leukemia cells can up-regulate the expression of CD15 differentiation antigen on the surface of K562, U937 and HL-60 cell membranes after administration of PSA (79). Another study showed that PSA can up-regulate the phosphorylation level of ERK and inhibit cell differentiation in the MEK/ERK pathway (49)

Similarly, the structure-activity and structure-toxicity relationship analysis of PSA derivatives have further confirmed that they also have anti-tumor effects. It inhibited tumor cell growth by causing G1 cell cycle stagnation (80).

#### Anemoside B4

In recent years, a large number of scientists have found that Anemoside B4 (AB4) had an anti-tumor effect. Xue et al. found that the inhibition rate of 100 μmol/L AB4 reached 46.26%. The mechanism may be related to up-regulation of the Bax/Bcl-2 ratio, activation of caspase-3, and cleavage of PARP (47). Besides, a study confirmed that AB4 inhibited the expression of Ki67 in tumor tissue, and its inhibitory effect on cancer may be connected with the expression of Notch signaling pathway-related proteins (51). Moreover, AB4 can promote the expression of autophagy-related molecules Beclin1 and Atg5, thereby inhibiting the proliferation and migration of human cervical cancer HeLa cells (52). Currently, recent studies reported that AB4 can down-regulate the expression of N-cadherin and up-regulate the expression of e-cadherin. The results illustrate that AB4 inhibited SKOV3 cell proliferation by regulating JAK/STAT3 signaling pathway-related proteins (50).

#### PC Saponins

PC saponin, a key class of compounds in PC, can inhibit the proliferation of tumor cells by down-regulating the expression levels of IL-6R, TNF-α and NF-KB in the inflammatory microenvironment of mice (41). Furthermore, PC saponins can inhibit the proliferation of HT29 cells in a dose-dependent manner (IC_50 =_ 1.440μg/ml). At the same time, it was detected to be related to the expression of Cleaved-caspase 3 (42). The main mechanism was that the expression levels of calpain1 and N-cadherin decreased, while E-cadherin increased gradually (43). In addition, research reported that the inhibitory rate of 25μg/ml PC saponins on the proliferation of 12 tumor cell lines was above 96%. The average IC_50_ range was 3.44-9.91μg/ml. For example, the IC_50_ values of PC saponins against human liver cancer cells (Bel-7402, SMMC-7721) and human colon cancer (HT29, HCT-116) were 5.12, 2.26, 2.03, and 3.24μg/ml, respectively (44). At present, some researchers have confirmed that after PC saponin administration, serum IL-6 and TNF-α levels, expression of VEGF and CD31 in liver tissues, and CXCR4, CXCL12, MMP-9 and MMP-2 proteins in spleen tumor tissues of colon cancer mice were significantly reduced. The mechanism may be related to the down-regulation of the CXCR4/CXCL12 signaling pathway in tumor tissues (81).

(Research Mechanism **Table 2**: 1-19).

### Induction Apoptosis

Apoptosis, which causes the initiative cell death process through the activation of a series of death signals, is quite different from cell necrosis (82). PC is reported to exhibit an anticancer effect *via* inducing apoptosis and its related cell death networks. The molecular mechanisms and biological functions are explained here.

#### PC saponin

A study showed that the apoptosis effect of tumor rats was verified by PC saponin administration and found no effect on the number of white blood cells, spleen and kidney indexes (83). In addition, PC saponins can significantly reduce the mitochondrial membrane potential, and up-regulate the expression of caspase-3, and caspase-9. The underlying molecular mechanisms involved in the activation of caspases were up-regulation of the pro-apoptotic proteins and downregulation of the anti-apoptosis protein Bcl-2 (56). Similarly, the level of Bax was also significantly increased (57). Meanwhile, PC saponins can also down-regulate the expression of the P I3K/AKT/m TOR signaling pathway-related protein PI3K-p85, p-AKT, p-mTOR, p-p70S6K protein (58, 59). In addition, PC saponins, in a time-dose-dependent manner, had a pro-apoptotic effect on multiple myeloma cells by up-regulating the expression of the antigen CD49e on the surface of multiple myeloma primary cells. At the same time, some cell growth was arrested in the G2 phase. It may be that BCL-2, cyclin-B1, and other proteins played a role in promoting apoptosis (60). Among them, PSD, R13, and PSA displayed greater antitumor activity (IC_50_ = 5.6, 5.1, and 10.5 µM, separately) against NCI-H460 cells compared with other monomers. In addition, through proteomics, DAVID Bioinformatics Resources, R software environment, and KEGG database analysis, candidate proteins (LDHA, PI3K, NOL3, and cleaved-caspase-3) may play a major regulatory role (53).

#### Pulsatilla Saponin D

PSD (20, 40 μmol/L) can induce apoptosis of lung adenocarcinoma cell A549 *in vitro*. Mechanically speaking, PSD can down-regulate BCL-2 and caspase-3 signal transduction. At the same time, PSD can also regulate the expression of proteins PI3K, p-Akt, p-m TOR, and p-p70S6K. Therefore, studies have shown that PSD can regulate the mitochondrial apoptotic pathway and PI3K/Akt/mTOR signaling pathway (54). Additionally, PSD strongly suppressed the growth of hepatocellular carcinoma cells and induced apoptosis by increasing the proportion of sub-G1 apoptotic cells from 8% to 21% through induction of expression of Bax and cleaved caspase-3 (55).

(Research Mechanism **Table 2**: 20-26).

### Blockage of Tumor Cell Cycle

The occurrence of tumors is associated with abnormal regulation of the cell cycle (84). Based on the existing literature, the molecular mechanism of blocking tumor cell cycle by PSA, AB4 and PC saponins was summarized.

#### Pulsatilla saponin A

Mechanically, p53 and cyclin B protein levels were higher, whereas Bcl-2 protein levels were lower in PSA–treated cancer cells (79). The results showed that PSA may induce DNA damage and G2 phase arrest of cancer cells to play an anti-tumor role (2).

#### Anemoside B4

Anemoside B4 had a significant inhibitory effect on HepG2, with the maximum inhibition rate reaching 71.5%. Through cell cycle distribution analysis, it was found that up to 83.2% of cells are blocked in the G2/M phase (2, 61). It has been reported that AB4 may significantly inhibit the proliferation and induce apoptosis of HepG2 cells and Huh-7 cells by regulating the Caspase 3 pathway (85).

#### PC Saponins

Total saponins from PC could make human liver cancer SMMC-7721 cells arrested in the S phase and human lung cancer A549 cells were arrested in G0/G 1 phase. At the same time, the protein expression levels of Cleaved PARP and Cleaved caspase-3 increased (62). Additionally, PC saponins induced significant G1/S depletion by increasing p21 and p27 mRNA and decreasing cyclin D1, cyclin-A, and cyclin E mRNA (63).

These studies suggested that the regulation of cell cycle-associated regulatory factors was one of the mechanisms of PC saponins in the prevention and therapeutic intervention of cancer (Research Mechanism **Table 2**: 27-28).

### Inhibition of Tumor Angiogenesis

Unrestricted invasive growth and metastasis of malignant tumors are all depending on vascular angiogenesis (86). Therefore, inhibition of tumor angiogenesis and blocking the angiogenesis pathway can effectively prevent the growth of the tumor (87). According to the existing literature, PSD is the main compound that PC plays an anti-tumor angiogenesis role. The specific mechanism is as follows.

#### Pulsatilla Saponin D

A study showed that PSD can significantly inhibit angiogenesis in mice with gastric cancer and effectively prolong the survival time of mice. Next, PSD showed a potent anti-angiogenic activity to decrease the expression of hypoxia-inducible factor-1α (HIF-1α) and vascular endothelial growth factor (VEGF) (64). Moreover, PSD was found to effectively suppress the phosphorylation of PI3K downstream factors, such as Akt, mTOR and p70S6K both *in vitro* and *in vivo*. It inhibited angiogenesis and induced apoptosis of hepatocellular carcinoma (55). And studies demonstrated that PSD inhibited the AKT/mTOR pathway, leading to the suppression of tumor growth and angiogenesis together with induction of apoptosis (88). Also, PSD docks at an allosteric site on mesenchymal-epithelial transition factor (c-Met) and thereby targets the c-Met signaling pathway to inhibit angiogenesis (65) (Research Mechanism **Table 2**: 29-31).

### Regulate Cell Energy Metabolism

Tumor cells need enough energy in order to reproduce indefinitely. This process is often through glycolysis to make the cells obtain enough energy materials (89). It has been reported that PC inhibits the energy metabolism of tumor cells mainly by regulating potential target proteins related to the glycolysis pathway. The specific mechanism is as follows.

#### PC Saponins

Studies have shown that PC saponins can significantly reduce glucose consumption, lactic acid production and adenosine triphosphate content in tumor cells (31). In addition, another study showed that PC saponins can reduce the key enzymes of glycolysis, such as hexokinase-II (HK-II), lactate dehydrogenase (LDHA), and M2 pyruvate kinase (PKM2), phosphofructokinase (PFK) and the amount of pyruvate kinase (PK) (67). At the same time, PC saponins can also increase the amount of succinate dehydrogenase (SDH) in the key enzymes of the tricarboxylic acid cycle (90).

At present, the existing literature shows that PC saponins can make glycolysis key proteins ERK1/2, hypoxia-inducible factor-1α (HIF-1a), Ras, glucose transporter 1 (GLUT1), lactate transporter (MCT4), Hexokinase 2 (HK2), MCT1, c-Myc and other factors were significantly reduced (91). The results showed that PC saponins can regulate the energy metabolism of tumor cells through the HIF-1α pathway to achieve the effect of inhibiting tumor growth (66). PC saponins may be a potential tumor energy metabolism blocker(Research Mechanism **Table 2**: 32-33).

### Reversal of Drug Resistance

Chemotherapy is a primary means of cancer treatment. However, multidrug resistance often occurs in tumor cells, reduces the efficacy of chemotherapy, and is the major cause for the failure of chemotherapy in cancer patients (92). Thus, it is urgent and important to improve drug resistance in cancer treatment. A study showed that Anemoside B4 decreased the expression of P-gp gene in human colon cancer L-OHP resistant cells LoVo/L-OHP (68). Furthermore, data were showing that PSD inhibited Met phosphorylation and downstream signaling pathways required for growth and survival in Met-expanded HCC827GR cells (93) (Research Mechanism **Table 2**: 34-35).

### Inducing Autophagy in Tumor Cells

Autophagy is an evolutionarily conserved mechanism to protect the cells from unfavorable environmental conditions (94). Inhibition of autophagy has been contemplated as a novel strategy to enhance the anticancer efficacy of existing chemotherapeutic agents (95).

#### *Pulsatilla* Saponin D

Zhang al et. indicated that PSD increased the phosphorylation of ERK and inhibited the phosphorylation of mTOR and p70S6K. This indicated PSD was an inducer of autophagosome formation (69). Notably, PSD significantly increased p62 protein levels. Simultaneously, the mechanistic study indicated that PSD profoundly abolished the co-localization of EGFP-LC3 and lysosomal-specific probe LysoTracker Red, suggesting that the autophagosome-lysosome fusion was blocked by PSD, which is similar to the action of chloroquine (70).

#### Anemoside B4

According to the existing literature, AB4 induces autophagy through four molecular pathways. 1)AB4 could regulate autophagy by acting on the Bcl-2-Caspase-3 and Beclin-1-LC3-P62 pathways. 2)AB4 could induce autophagy by up-regulating the expression of LC3-II/LC3-I and Beclin-1 protein and down-regulating the expression of p62 protein (71, 96). 3)AB4 may down-regulate the expression of P-Akt/Akt and P-MTOR/mTOR proteins in PI3K/Akt/mTOR signaling pathway and induce autophagy in SMMC7721 cells (72). 4)AB4 could inhibit Notch signaling and promote the expression of autophagy related molecules Beclin1 and Atg5 to induce autophagy (34).

Collectively, the study highlighted PSD and AB4 might be a novel way to treat carcinoma (Research Mechanism **Table 2**: 36-38).

## Clinical Application

At present, after treatment with PC compound, adverse reactions such as neutropenia, vomiting and diarrhea were improved in 66 patients (97). It is reported that this may be related to the inhibition of zDHHC9 expression and the restricted function of the Ras gene (98). In addition, studies have shown that the TCM compound mainly based on PC is also used to treat complications caused by radiotherapy in 22 patients with cervical cancer (99). In short, the TCM compound mainly based on PC has achieved good clinical effects in the adjuvant treatment of colorectal cancer patients.

## Pharmacokinetic Studies

Pharmacokinetic studies on PC are vital to clarify the principle of compatibility. The mechanisms contribute to deciphering a reasonable dose and lower clinical adverse reactions. Studies have shown that the main part of intestinal absorption of PC saponin is the duodenum (100). Simultaneously, the efficacy of PC saponin is concentration-dependent (101).

### *Pulsatilla* Saponin D

In the last several years, studies have developed an LC-MS/MS method to determine the pharmacokinetics and oral bioavailability of PSD in rats (102). Interestingly, although the oral bioavailability of PSD was confirmed to be less than 5%, the efficacy experiments showed that the anti-tumor activities of PSD were particularly obvious (103). In addition, Rao et al. identified 18 metabolites of PSD in rat plasma, urine, and stool samples (104). It has been confirmed that the metabolic process of PSD in the body was mainly through glycosylation, deglycosylation, dehydrogenation, hydroxylation, and sulfation (105). Similarly, Yan et al. confirmed that the metabolic rate of PSD in the intestinal flora of liver cancer patients was significantly lower by collecting fresh feces from liver cancer patients and healthy people. In humans, this is the same rate as ginsenoside Rh2 in the intestinal flora of liver cancer patients (106). This showed that PSD can obviously play an anti-cancer effect. However, the factors that played a role in the pharmacokinetics of PSD needed to be further studied.

### Anemoside B4

AB4 (50 mg/kg) was reported to be rapidly cleared from rat plasma. At the same time, the plasma T_1/2_ of the rats in each group was 1.18 and 1.40 h after i.p. and i.v. administration, respectively. The study also showed that AB4 was widely distributed in various tissues after injection. Among them, the highest concentration of AB4 in the kidney (≈4,800 ng/g) was found at 0.5 h after i.v. injection (107). This result indicated that the kidney may be a target organ for AB4. Meanwhile, other researchers found that AB4 (6 mg/kg) was released in the lungs at a concentration of 125.5μg/g after 24 h of intratracheal instillation (108). This study demonstrated that AB4 can accumulate in the lungs and then be slowly released into the bloodstream, extending its retention time in the body. Additionally, an anti-tumor assay showed that the metabolite pool had stronger activity in decreasing cell viability of human HCC SMMC-7721, HeLa and MCF-7 cell lines than did AB4 itself (109, 110). The results suggest that AB4 may exert antitumor effects through its active metabolites.

### Others

In addition, studies have shown that the permeability coefficients of Pulsatilla saponins B3, BD, B7, B10, and B11 in different intestinal segments were absorbed as duodenum> jejunum> colon> ileum (111). Among them, the pharmacokinetic study of the main active ingredients in PC was shown in **Table 3** (112). PC were quickly absorbed by intragastric administration in rats, eliminated quickly, and have low absolute bioavailability. The pharmacokinetics of PC confirmed its broad application prospects in development and utilization.

**Table 3:** The pharmacokinetic study of the main active ingredients.

	Pharmacokinetics				Intestinal degradation kinetics		
chemical composition	Intragastric administration (Tmax)	Intragastric administrati on (T1/2)	Intravenous administrati on (T1/2)	Bioavailability (%)	Degradation rate constant (KA)	Valid period (t0.9)	Half-period (1/2)
Pulsatilla saponinsB3	0.33	12.81	0.43	1.16	0.0794	1.327	8.73
Pulsatilla saponinsBD	0.37	18.52	2.23	1.17	0.0523	2.015	13.25
Pulsatilla saponinsB7	0.51	16.31	0.56	0.55	0.0539	1.955	12.86
Pulsatilla saponinsB10	0.51	7.03	0.35	0.96	0.0426	2.473	16.27
Pulsatilla saponinsB11	0.51	12.54	0.34	2.5	0.0468	2.251	14.81

## Bioinformatics

Bioinformatics is a common method to study the multi-target characteristics of TCM. It was reported that 11 active compounds in PC were found to overlap with colorectal cancer target genes through network pharmacology analysis. Among them, 21 core genes play an anticancer role through 11 major pathways including the p53 signal transduction pathway and the central carbon metabolism pathway in cancer. Furthermore, molecular docking results showed that the saponins of PC had a good binding effect on the target genes of colorectal cancer. Among them, PSA can bind to colorectal cancer proteins HSP90, KIT, and SIRT1 (113).

In addition, some researchers explored the bioinformatics of Baitouweng Decoction, a TCM compound based on PC. It is reported that 5 cases of colon cancer patients were purified by blood samples before and after treatment with Baitouweng Decoction. Agilent chip and computer software were used to screen 159 differentially expressed genes, including 38 up-regulated genes and 121 down-regulated genes. It is mainly manifested in Jak-STAT, MAPK, and other tumor-related signaling pathways (114). Another study predicted by network pharmacology that Baitouweng decoction may affect tumor cell proliferation, migration, apoptosis, and angiogenesis by regulating signaling pathways such as TNF, PI3K-Akt, HIF1, and vascular endothelial growth factor (115). These results provide a reference for discussing the anti-tumor mechanism of PC.

## Conclusion

In this review, we summarized the reports on the anticancer effect and molecular mechanisms of active compounds and extracts of PC in both laboratory and clinic. In recent years, the anticancer effect of PC extracts against liver, lung, breast, gastric, and colon cancer, etc has been widely investigated. The majority of the scientific literature reported that PC exerted anticancer effects through suppressing cell proliferation, inducing apoptosis, inhibiting migration, inhibiting invasion, inducing autophagy, and restraining angiogenesis. To determine the molecular mechanism of the anticancer effect of PC, detailed studies have been carried out. The reported molecular mechanisms include Notch, PI3K/AKT/mTOR, AKT/mTOR, and MEK/ERK signaling pathways. PC can down-regulate the expression levels of IL-6R, TNF-α, NF-kB, Bcl-2, and Caspase-3, meanwhile, up-regulate the expression of autophagy-related molecules Beclin1 and Atg5. In addition, PC can reduce the expression of HIF-1α and VEGF and effectively inhibit the phosphorylation of PI3K downstream factors (such as Akt, mTOR, and p70S6K). Finally, it induces tumor cell apoptosis by inhibiting angiogenesis. Interestingly, PC regulates energy metabolism of tumor cells by reducing glucose consumption, lactic acid production and adenosine triphosphate content. At the same time, PC can also down-regulate key glycolytic proteins GLUT1, HK2, MCT4, MCT1, c-Myc and HIF-1a. It can be seen from the literature that the curative effect of TCM and the molecular mechanisms involved are very complicated. Although the biological role of the anticancer activity of PC has been reported in detail, the mechanisms of anti-tumor effects of PC saponins remain to be further identified. More explorations remain to be performed, such as the effects of PC saponins on cancer metastasis or immunity. It may be hoped that further studies will be conducted on PC saponins to identify more effective anti-tumor components.

PC extract has been widely applied in China as an alternative medicine against diseases. This review provides the latest progress in the anti-tumor activity and mechanism of PC. At the same time, the clinical application, pharmacokinetics and bioinformatics of PC are also described. Among them, the compounds PSA, PSD, AB4, and R13 have high anti-tumor activity. Some studies have confirmed that the anti-tumor effect of R13 is comparable to that of cisplatin (53). Meanwhile, the effect of R13 on apoptosis was stronger than that of PSD. However, as far as the existing literature is concerned, there are few significant antitumor studies of R13. Therefore, it is imminent to study the molecular mechanism of the antitumor effect of active compounds in PC. In addition, it is an important research topic to find the optimal process conditions for the further separation and purification of PC saponins monomer.

Moreover, studies based on UPLC-QTOF-MS serum metabolomics studies have confirmed that long-term oral PC total saponins can cause chronic liver damage, and its safety needs further attention (116). To be sure, the development of highly efficient and low toxic anti-cancer drugs is one of the most urgent problems in the medical field. PC compounds have the characteristics of natural, low toxicity, and high efficiency, which allows them a promising antitumor drug.

## Author Contributions

HL wrote the full article and provided ideas. Meanwhile, according to the requirements of editors and reviewers, HL revised the whole process. LW provided the idea for the article. XZ modified the language of the article. WX and XZ modified **Figure 2**. HS and XF conducted the article for guidance. All authors contributed to the article and approved the submitted version.

## Funding

Study on the material basis and mechanism of effect of national medicine Thymus quinquecostatus on ischemic cardio-cerebrovascular diseases; National Natural Science Foundation of China(81760769); Study on anti-reperfusion injury metabolism of polyphenols extracted from Chinese medicinal herb Thymus quinquecostatus; Key research and development plan of autonomous region(2020BFG03007); Special Talents Initiation Project of Ningxia Medical University(XT2013003).

## Conflict of Interest

The authors declare that the research was conducted in the absence of any commercial or financial relationships that could be construed as a potential conflict of interest.

## Publisher’s Note

All claims expressed in this article are solely those of the authors and do not necessarily represent those of their affiliated organizations, or those of the publisher, the editors and the reviewers. Any product that may be evaluated in this article, or claim that may be made by its manufacturer, is not guaranteed or endorsed by the publisher.

## Abbreviations

PC saponins, Pulsatilla chinensis saponins; TCM,Traditional Chinese medicine; PSA, pulsatilla saponin A; AB4, Anemoside B4; R13, raddeanoside R13; PSD, Pulsatilla saponins D; 5−FU, fluorouracil; HIF-1α, hypoxia-inducible factor-1α; VEGF,vascular endothelial growth factor; c-Met, c-mesenchymal-epithelial; HK-II, hexokinase-II; PFK, phosphofructokinase; PK, pyruvate kinase; P-gp,P-glycoprotein.
